# Simultaneous Bilateral Avulsion of Tibial Anterior Tubercle in Adolescent: Two Case Reports and Narrative Review of the Literature

**DOI:** 10.1155/2023/1035705

**Published:** 2023-02-09

**Authors:** Dario Giunchi, Jorge Gonzalez, Marco Odorizzi, Mario Sagaon Mendoza, Vincenzo De Rosa

**Affiliations:** ^1^Servizio di Chirurgia e Ortopedia, Unità Traumatologia e Ortopedia, Ospedale Regionale di Lugano, Ente Ospedaliero Cantonale, Ticino, Switzerland; ^2^Clinica di Chirurgia e Ortopedia Pediatrica IPSI, Ente Ospedaliero Cantonale, Ospedale San Giovanni di Bellinzona, Ticino, Switzerland; ^3^Universitäts-Kinderspital beider Basel, Basel, Switzerland

## Abstract

Fractures of the anterior tibial tuberosity are uncommon, ranging from 0.4% to 2.7% of all epiphyseal lesions reported. Bilateral sequential fractures are even rarer, with less than twenty-eight cases described to date and, as such, there is very little consensus data on their treatment as a whole. We report the first two documented cases of bilateral tibial tuberosity avulsions of the anterior tubercle in Switzerland, treated by open reduction and internal fixation. Both cases were 14-year-old healthy males with no previous medical history, who both suffered extra-articular fractures after falling from a height. The first case was treated in 2012 with a four-year long clinical follow-up and full recovery. The second, treated in 2019, was clinically followed for one year with a complete recovery and has returned to sporting activity at a pre-injury level. Due to the rarity of the condition, a lack of consensus on the optimal therapy, we believe the documentation of these two cases treated by the same team may be of clinical relevance.

## 1. Introduction

Avulsion fractures of the anterior tibial tubercle represents 3% of all injuries of the proximal tibia and 0.4–2.7% of all epiphyseal fractures [1]. Bilateral simultaneous avulsions of the anterior tibial tuberosity are extremely rare with the first case described by Borsch Madsen [2]. Since then, less than 28 cases have been reported in modern literature. Most recent studies reports a variable number of cases ranging from 15 [3] to 21 [4], but in nearly all cases, the patient is a young male teenager, with the mean age being 14 years old as reported by Shuvendu et al. [5] in their review of the last sixty years of the literature on tibial avulsions. The two cases we illustrate in our study follow this trend and correspond to the most common mechanism of injury in this patient group.

The first classification of this fracture was provided by Watson-Jones [6] in 1976 with consideration to three fracture types. Since then, various modifications have been proposed.

As reported by McKoy and Stanitski on analysis of the small case series to date, several methods of fixation are possible, but the most commonly used is the tension band wiring or cannulated screws [7].

Nevertheless, due to the scarcity of these lesions there is not an overall agreement on the best diagnostic classification and no consensus on the gold standard of treatment and consequential outcomes. The aim of this study is to disseminate relevant clinical and epidemiological data with regard to the first reported cases in Switzerland of this pathology.

## 2. Case Report I

**Figure 1:**
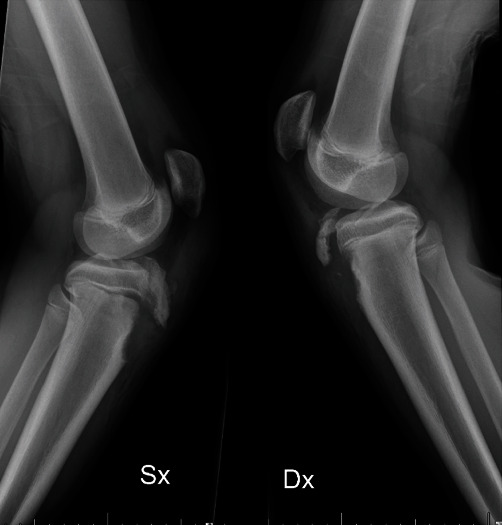
Case I: X-ray imaging showing asymmetrical bilateral tibial tuberosity avulsions with a type IIB of the right knee and type III B of the left, without intra-articular involvement.

The first case reported in 2012, involved an active 14-year-old male, with a healthy body mass index, who after falling from a height, and landed with both feet on a hard flat surface. The male reported immediate bilateral knee pain with functional impairment. Clinical evaluation showed bilateral knee swelling, limited passive movement, and in the absence of other post-traumatic pathologies. Radiographs imaging (Figure 1) showed a type IIB right knee fracture and a type IIIB left knee fracture according to the Watson-Jones classification modified by Ogden et al. [8].

**Figure 2:**
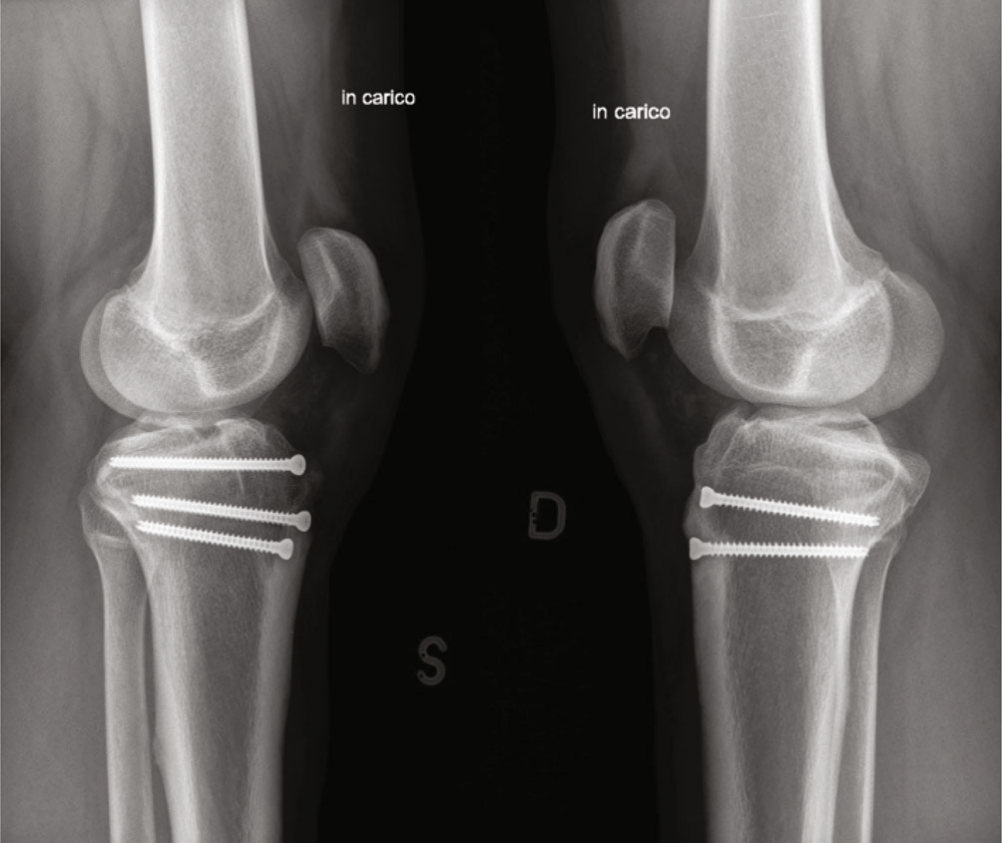
Post-operative imaging at 4 months demonstrating anatomic reduction and fixation with cannulated full threaded 4.5 mm screws.

Within forty-eight hours of diagnosis, the patient underwent bilateral open reduction and internal fixation (ORIF; Figure 2). On the left-hand side, we placed three cannulated fully threaded 4.5 mm screws, one proximal and two distal to the tibial tubercle. On the right-hand side, two identical 4.5 mm screws were placed distal to the knee physis.

Post-operative therapy included 4 weeks without weight bearing, full lower limb casts followed by gradual and complete restoration of joint range-of-motion (ROM) and weight bearing by the fifth week post-operative under physiotherapeutic supervision. Full subjective and clinical recovery with complete ROM and normal muscle strength were achieved by the 16th post-operative week, with full return to normal activities of daily living. At follow-up evaluation 4 years postoperatively, there were neither negative outcomes nor need for hardware removal.

## 3. Case Report II

**Figure 3:**
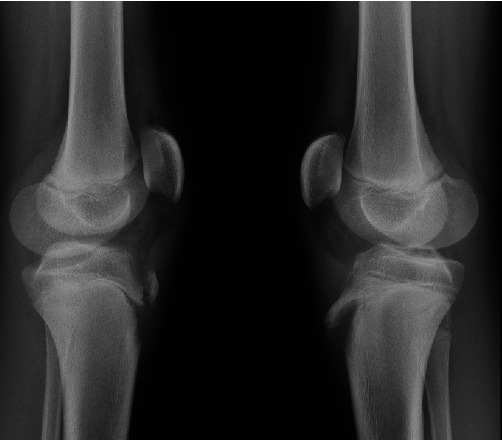
Case II: X-ray imaging asymmetrical avulsion fractures with a type IIA of the right knee and type II B of the left, again without intra-articular involvement.

The second case occurred in 2019. As in the first case, a previously healthy, normal weight, 14-year-old male, who played school-level competitive basketball, presented to our Emergencies (A&E) after jumping from ground level and landing on a hard flat surface. The symptoms and signs were identical to the first case with immediate pain and ROM limitation. Clinical evaluation found both knees swollen, with minimum possibility of passive movement and inability to weight bear. X-rays (Figure 3) showed a type IIA right knee avulsion fracture and a type IIB left knee avulsion fracture according to the Ogden modified classification.

**Figure 4:**
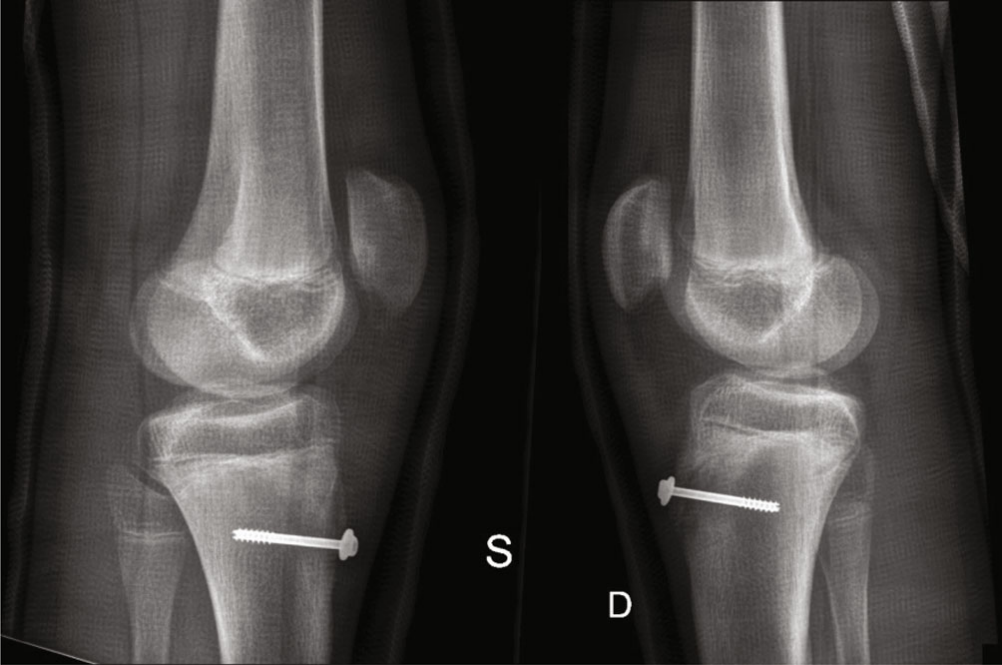
Showing case II post-operative result at week 1.

**Figure 5:**
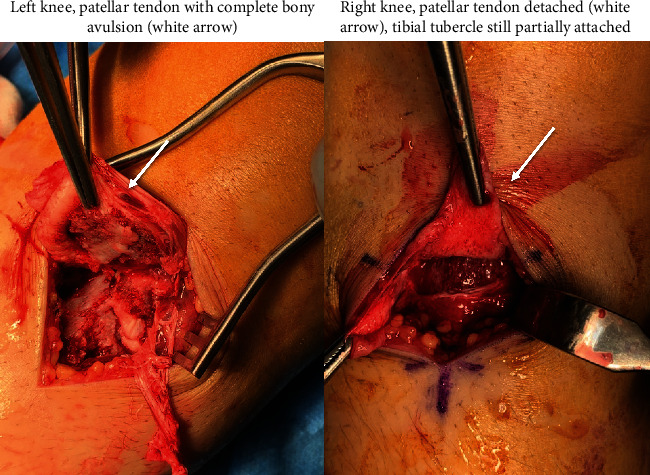
Photographic images demonstrating complete patellar tendon distal detachment.

The patient underwent surgery and had bilateral ORIF repair. For both knees, the fracture sight was debrided allowing for anatomical reduction, and osteosynthesis was achieved using the same hardware: one 4.5 mm partially threaded cannulated screw (Figure 4) placed distally to the physics with washer and precise reconstruction of the surrounding soft tissues. Intraoperatively complete detachment of the patellar tendon was documented for both knees (Figure 5) that has been treated by direct suturing.

In the case of the second male, post-operative care involved two full lower limb casts, no weight bearing for six weeks, followed by a gradual increase of knee flexion and weight bearing under physiotherapist guidance. Full subjective and clinical recovery with complete ROM were achieved by post-operative week 12 with full return to normal activity. Light on-field workouts were started at week 13, the first competitive sporting event at week 20, and return to pre-injury level was achieved at six months postoperatively. At a distance of one year, clinical and radiological evaluation confirmed a successful outcome and there was no need for hardware removal.

## 4. Discussion

Bilateral simultaneous avulsions of anterior tibial tubercle are very uncommon. As stated in the introduction less than twenty-eight cases are described to date, but it is reasonable to assume that not every case that has been diagnosed or treated worldwide is reported in the literature. With that in mind, one may presume that the condition may be more common than that reflected in the literature; however, further identification and pathophysiological investigation and case dissemination are necessary before a true epidemiology understanding may be achieved. Roy and Nag reported a mean age of 14 years in an almost entirely male population, close to 97% of cases [5], and our patients reflect this demographic data.

Nicolini et al. [9] described that the anterior part of the proximal tibia is a region of weakness persisting longer than the posterior part of the tibia during the osteogenesis and this explains the risk of avulsion. This statement reflects the findings of Ehrenborg and Lagregren who divided the tibial tubercle development into four stages [10]: cartilaginous, apophyseal, epiphyseal, and bony. Uniquely during the epiphyseal stage, do the secondary ossification centers form an anterior bony tongue that represents the probable point of greatest weakness. The entire process that gives mechanical strength to the patellar tendon attachment is generally complete in females by the age of 15 years and in males by the age of 17 years [11]. This anatomical development variation, in combination with a consistently higher athletic participation in males, partially explains the prevalence of gender divergence in the epidemiological data gathered to date.

Traumatic tibial tubercle avulsion in these population groups may occur due to two possible mechanisms linked to the activity of jumping. As reported by Hanley et al. [12], the take off phase involves a powerful contraction of the quadriceps during knee extension and, subsequently, while landing, a rapid eccentric knee flexion, which opposes the contracted quadriceps. In our two cases, landing after jumping is the lesions causative aetiology.

These mechanical vector forces in concert with the epiphyseal developmental weakness located at the anterior tibial tubercle are the probable cause of the “classical” tibial tubercle avulsion in this young population group.

Several co-morbidities may be related to anterior tubercle avulsion, such as Osgood Schlatter disease, osteogenesis imperfecta, and vitamin D deficiency [13, 14]. Our patients were completely healthy individuals and as we had no reason to suspect a metabolic impairment, we did not test their vitamin D blood levels.

Watson-Jones first introduced the classification in 1955 describing three types of fractures [6], followed by Ogden et al., who modified the system through comminution and displacement adding A and B types in 1980. Further sub types were introduced in 1985 by Ryu and Debenham [15], with the addition of a type IV (propagation of the fracture line into the posterior cortex). The latest review in 2003 has been provided by McKoy and Stanitski who added the classification modification of a type V also called type Y [7], which is a combination of type III and IV as visible in Table 1. In our cases, we had two type IIIB fractures, one IIB and one IIA in our case population following Ogden et al.

As reported by Roy and Nag [5], which is the most detailed review so far, the majority of previous cases where type III (20 knees) according to Ogden classification (modification of Watson-Jones), 8 knees were type II, 6 type IV, 2 type I, and no data for 6 knees. All of them except four were treated with ORIF. Therefore, our cases are in line with these findings.

Anterior tibial avulsion may be associated (1–2%) with collateral and cruciate ligament injuries, meniscal tears, patellar or quadriceps tendon tears, and joint cartilage lesions, especially in the case of intra-articular involvement. Moreover, Ivan Yue et al. [18] report that compartment syndrome of the anterior compartment may occur in up to 10–20% of cases [18].

According to Elbaum [16] ORIF should be the standard approach for grade higher than II (Watson-Jones classification revised by Ogden, Ryu, and McKoy). Closed reduction should be attempted only in a grade I avulsion. We treated our patients both with ORIF, but the choice of the implants and post-operative management were dependent on operator's preferences, type of fracture, and patient's functional requirements. As reported by McKoy on analysis of the small case series to date, several methods of fixation are possible, but the most commonly used is the tension band wiring or cannulated screws.

The average time of patient's return to full activity (e.g., sport) ranges between 4 months and 1 year, but complications, such as knee rigidity, genu recurvatum, and patellar malposition are possible documented complications. Non-union, malunion, bursitis, and infection are also reported [1]. Our first patient had a complete recovery and returned to normal activity within 4 months, but did not practice sport. Our second patient is a basketball player, who returned to a pre-injury sporting level at 6 months postoperatively. Juan Pretell-Mazzini et al. in their literature review stated that general outcome of single fracture is excellent with 98% of patients able to return to pre-injury activities. To date there is a scarcity of published outcomes on bilateral tibial avulsion with regards to monitored physical activity following bilateral osteosynthesis.

Borsch-Madsen first described this injury in 1955 [2]. We conducted a search of PubMed using the following search combinations “tibial AND (tubercle OR tuberosity) AND (fracture OR avulsion) AND (simultaneous OR bilateral OR combined).” One hundred and twenty seven articles were found and the latest complete review of the literature has conducted by Roy and Nag [5] in 2013 reporting twenty-one cases. Since then, we can add one case by Khoriati et al. [17], one case by Nicolini et al. [9], one case by Yue et al. [18], and four cases by Fernandez Fernandez et al. [19]; therefore, amounting to twenty eight reported cases. Our two cases would be the 29th and 30th reported and the first two documented in Switzerland. All the patients were aged from 13 to 17 years old, all except one were male, all suffered the injuries while jumping or landing or running, all were treated with ORIF except for three cases, where a closed anatomical reduction was performed and another that was treated conservatively by immobilisation whose reduction has been performed close and one treated with only casting. All the patients had an excellent functional long-term outcome. Complications, such as premature closure of epiphyses, flexion deformity, and compartment syndrome were encountered in four cases. Removing of the implanted hardware was performed in three reported cases.

## 5. Conclusion

We reported the first two documented cases of traumatic bilateral simultaneous tibial tuberosity avulsion in Switzerland. Despite the rarity of this pathology, our two cases have been treated similarly to all the other known and reported cases with positive outcomes. Indeed, due to the rarity of the condition, a lack of consensus on a standardized therapy, we believe the documentation and dissemination of our two cases treated by the same team may be of clinical relevance.

## 6. Clinical Message

Simultaneous bilateral avulsion of tibial anterior tubercle in adolescent is a rare condition, and there is a lack of consensus on the optimal therapy, we showed how two clinical cases have been managed.

## Figures and Tables

**Table 1:** Anterior tibial tubercle fracture avulsion.

Type I	Small fragment of the tubercle is avulsed and displaced proximally
	Subtype A: undisplaced
	Subtype B: displaced or comminuted
Type II	The secondary ossification center has already fused with the proximal tibia epiphysis, and the entire tubercle is displaced proximally
	Subtype A: undisplaced
	Subtype B: displaced or comminuted
Type III	Fracture passing across the proximal physis into the joint, involving part of it
	Subtype A: undisplaced
	Subtype B: displaced or comminuted
Type IV	Fracture passing entirely through the proximal physis without involving the joint
	Subtype A: undisplaced
	Subtype B: displaced or comminuted
Type V	Type III with fracture involving the remaining part of the physis, usually the posterior part
	Subtype A: undisplaced
	Subtype B: displaced or comminuted

Note. Watson-Jones classification modified by Ogden, Ryu, and Mckoy. (1) Type I. (2) Type II. (3) Type III with intra-articular extension. (4) Type IV with entire proximal tibial physeal separation. (5) Type V resembling a combination of types IIIb and Salter Harris type IV.
